# Formulations of poly(vinyl alcohol) functionalized silk fibroin nanoparticles for the oral delivery of zwitterionic ciprofloxacin

**DOI:** 10.1371/journal.pone.0306140

**Published:** 2024-08-01

**Authors:** Nguyen Thi Phuong Thao, Ngoc Yen Nguyen, Van Ben Co, Luong Huynh Vu Thanh, Manh Quan Nguyen, Suchiwa Pan-On, Duy Toan Pham

**Affiliations:** 1 Faculty of Health Sciences, Dong Nai Technology University, Bien Hoa City, Vietnam; 2 Department of Health Sciences, College of Natural Sciences, Can Tho University, Can Tho, Vietnam; 3 Faculty of Chemical Engineering, College of Engineering, Can Tho University, Can Tho, Vietnam; 4 Department of Analytical Chemistry-Drug Quality Control, Faculty of Pharmacy, Can Tho University of Medicine and Pharmacy, Can Tho, Vietnam; 5 Faculty of Pharmaceutical Sciences, Burapha University, Chonburi, Thailand; Hamadan University of Medical Sciences, ISLAMIC REPUBLIC OF IRAN

## Abstract

Fibroin nanoparticles (FNP) have been employed in numerous biomedical applications. However, limited research has focused on the oral delivery of FNP and in-depth molecular interactions between the encapsulated drug and FNP. Therefore, this work developed the FNP, functionalized with poly(vinyl alcohol) (PVA), to orally deliver the zwitterionic ciprofloxacin, focused on the molecular interactions. The particles were formulated using both desolvation (the drug precipitated during the particles formulation) and adsorption (the drug adsorbed on the particles surfaces) methods. The optimal formula possessed a size of ~630 nm with narrow size distribution (measured by DLS method), spherical shape (determined by SEM), and moderate drug loading (confirmed by FT-IR, XRD, and DSC techniques) of ~50% for the desolvation method and ~43% for the adsorption method. More than 80% of the drug molecules resided on the particle surfaces, mainly via electrostatic forces with fibroin. The drug was physically adsorbed onto FNP, which followed Langmuir model and pseudo second-order kinetics. In the in-vitro simulated gastric condition at pH 1.2, the ciprofloxacin bound strongly with FNP via electrostatic forces, thus hindering the drug release (< 40%). Contrastingly, in the simulated intestinal condition at pH 6.8, the particles could control the drug release rates dependent on the PVA amount, with up to ~100% drug release. Lastly, the particles possessed adequate antibacterial activities on *Bacillus subtilis*, *Escherichia coli*, and *Salmonella enterica*, with MIC of 128, 8, and 32 μg/mL, respectively. In summary, the FNP and PVA functionalized FNP could be a potential oral delivery system for zwitterionic drugs.

## Introduction

Silk, generally extracted from the *Bombyx mori* silkworm, has a rich history in biomedical applications [1]. A silk fiber composes of an outer layer of sericin (~30% w/w) and an inner core of fibroin (~70% w/w), of which the fibroin is a protein that has been approved by the United States Food and Drug Administration as a biomaterial in 1993 [2]. Fibroin consists of ~5,507 amino acids (mostly glycine, alanine, and serine) that are separated into two chains, the heavy chains and the light chains, linked to each other via a disulfide bond [3]. Fibroin inherent amphiphilic structure allows it to self-restructure into micro-/nanoparticles with little or no catalysis [4]. Due to this unique property, as well as the biodegradability [5], biocompatibility [6], and non-cytotoxicity [7], fibroin has been increasingly utilized as nanoparticulate drug delivery systems in numerous pharmaceutical products for injection [8, 9], topical [10], oral [11, 12], and ocular [13] administrations. Most studies developed fibroin nanoparticles (FNP) to deliver different drugs with small/medium/big molecular structures, hydrophilic/lipophilic properties, and charged/non-charged characteristics. Nevertheless, to the best of our knowledge, no research has been focused on the in-depth molecular interactions between the encapsulated drug and the FNP, thus limiting the current knowledge on how the drugs bind to and release from the FNP.

The interactions between an encapsulated moiety and the carrier nanomaterial are the main factors governing the drug behaviors (i.e., drug loading, drug release rates, drug target-binding efficacy) in both in-vitro and in-vivo biomedical settings [14–17]. This fact is even more crucial in oral administration, in which the gastrointestinal pH varies significantly from the stomach (pH ~ 1–2), the small intestine (pH ~ 6–7), to the colon (pH ~ 7–9). For instance, in our previous work, the chitosan functionalized fibroin films, which possess both negative charge domains of fibroin and positive charge domains of chitosan, could successfully load the zwitterion rhodamine B and the negatively charged 5(6)-carboxyfluorescein via ionic interactions, but not the non-charged acetaminophen [18]. Moreover, the drug loading amounts and their respective release rates were strongly dependent on the drug pKa and the medium pH (factors that affect the drug ionization states, based on Henderson-Hasselbalch equation) [18, 19]. However, in-depth effects of the drug-fibroin interactions on the drug adsorption and release profiles are unknown.

Ciprofloxacin (CIP) is a fluoroquinolone antibiotic used to treat bacterial infections, especially drug-resistant bacteria, by reducing DNA replications via inhibitions of bacterial DNA gyrase and topoisomerase IV [20–22]. In aqueous solution, depending on the pH, CIP is an amphoteric molecule that could be charged (positive charge due to the protonated amine groups at pH < 6 and negative charge due to the deprotonated carboxyl group at pH > 9) and non-charged/zwitterionic (at neutral pH) [23]. Consequently, CIP is extensively soluble at acidic/basic pH and sparingly soluble at neutral pH [24]. Numerous materials have been evaluated regarding the CIP adsorption/release profiles and their mechanisms/kinetics, namely natural bio-sorbents, magnetic materials, clays, hydrogels, and activated carbons [25–28]. Nevertheless, most studies tackled the environmental aspects of removing CIP as a pollutant, but not the pharmaceutical aspects of adsorbing and releasing CIP in a drug delivery system.

For those aforementioned reasons, this work formulated the FNP, without or with the poly(vinyl alcohol) (PVA) functionalization, for the oral delivery of the zwitterionic CIP, focusing on the drug/fibroin interactions, drug adsorption, and drug release mechanisms.

## Materials and methods

### Materials

The *Bombyx mori* silkworm cocoons, M45 species, were obtained directly from farmers in Truc Ninh, Nam Dinh, Vietnam, and transferred to the laboratory by specialized container trucks. The cocoons were kept at room temperature in zipper packs until uses. CIP, PVA, and dialysis tubing cellulose membrane (12000 MWCO) were provided by Sigma-Aldrich, Singapore. Sodium carbonate (Na_2_CO_3_), hydrochloric acid (HCl), calcium nitrate (Ca(NO_3_)_2_), calcium chloride (CaCl_2_), disodium phosphate (Na_2_HPO_4_), monopotassium phosphate (KH_2_PO_4_), sodium chloride (NaCl), and ethanol 99% were supplied by Xilong, China. All other chemicals/solvents were of reagent grades or higher.

### Fibroin extraction

The fibroin extraction process was conducted following the published reports [29, 30]. Initially, to remove the sericin outer layer, the silkworm cocoons (10 g) were immersed in 100 mL of Na_2_CO_3_ 0.5% (w/w) solution at 80–100°C for 1 h. The sericin-free silk strands were then washed with water, air-dried at room temperature, and dissolved in the strong salt mixture of CaCl_2_:Ca(NO_3_)_2_:EtOH:H_2_O at a weight ratio of 30:5:20:45. Next, the fibroin solution was dialyzed at room temperature for 3–5 days using a cellulose membrane (12,000 MWCO), centrifuged at 6,000 rpm, 20 min (EBA 20, Hettich, Germany) to remove the residues, and the clear stock fibroin solution was kept at 4°C for further experiments [3]. To determine the fibroin concentration, the UV-Vis spectroscopy was employed, with an absorbance wavelength of 276 nm, and the fibroin standard curve in water (y = 1.1517x + 0.0099, R^2^ = 0.9999). The extracted fibroin compositions and properties have been critically characterized in our previous work [30]. This simple extraction process, which only employed water, ethanol, and common inorganic salts of Na_2_CO_3_, CaCl_2_, and Ca(NO_3_)_2_, as well as standard laboratory techniques (i.e., mixing, heating, dialysis, and centrifugation), could be considered environmental friendly and safety to the technicians.

### Formulations of the blank FNP and PVA functionalized FNP

Prior to the formulations of the CIP loaded FNP, the blank FNP and PVA functionalized FNP (FNP/PVA) were prepared by the simple desolvation method [8], with varied factors of fibroin:ethanol ratios (v/v) and PVA concentrations. For this, the stock fibroin solution was diluted with water to a 1% (w/v) solution. Then, PVA solution was mixed with fibroin solution to get the final PVA concentrations (w/v) of 0% (for the FNP formula), 1% (FNP/PVA 1), 3% (FNP/PVA 3), and 5% (FNP/PVA 5). Next, ethanol 96% (v/v) was added dropwise onto the mixtures at different fibroin:ethanol ratios of 1:1, 1:2, 1:3, 1:5, and 1:7 (v/v). Finally, the mixtures were shaken for 1 h and cold centrifuged at 18,000 rpm for 30 min (Mikro 220R, Hettich, Germany) to receive the nanoparticles. The obtained particles were washed with water by centrifugation technique, lyophilized (FreeZone, Labconco, USA), and kept at 4°C until uses.

### Formulations of the CIP loaded FNP and CIP loaded FNP/PVA

The CIP loaded FNP (FNP-CIP) and CIP loaded FNP/PVA (FNP/PVA-CIP) were prepared by two different methods, the standard desolvation method and the adsorption method [12]. Both methods were mild and straight-forward, of which the desolvation process was the simple mixing of the water phase and the ethanol phase, whereas the adsorption process was the immersion of nanoparticles in the drug solution. Additionally, without the utilization of organic solvents, the methods were safe and environmental friendly.

#### Desolvation method

For this, the process was similar to that of the blank particles. Briefly, for each formula, 500 μg or 1,000 μg pure CIP was dissolved in the ethanol phase, and this phase was added dropwise into the fibroin/PVA solutions (PVA 0%, 1%, 3%, and 5%) with fibroin:ethanol ratio of 1:5 (v/v) (the optimal ratio). The mixture was then shaken at 200 rpm for 1 h at room temperature and cold centrifuged (4°C) at 18,000 rpm for 30 min (Mikro 220R, Hettich, Germany) to receive the nanoparticles. The obtained particles were washed with water by centrifugation technique, lyophilized (FreeZone, Labconco, USA), and kept at 4°C until uses.

The free-CIP in the supernatant, after particle centrifugation, was determined by UV-Vis spectroscopy (Jasco V730, Jasco, Japan) at a wavelength of 278 nm. The unloaded CIP amount was then determined based on the CIP standard curve (y = 0.1141x + 0.0551, R^2^ = 0.9994) and the drug entrapment efficiency (%) (EE%) was calculated using Eq (1).


$$\%\;CIP\;entrapment\;efficiency=\frac{Initial\;CIP\;amount-Unloaded\;CIP\;amount}{Initial\;CIP\;amount}\times 100$$ (1)

#### Adsorption method

For this method, 15 mg of the lyophilized blank FNP and FNP/PVA were homogenously dispersed in 50 mL CIP solution (10 μg/mL in water pH 6.2), followed by magnetic stirring at 200 rpm for 4 h at room temperature. At each time point of 30, 60, 90, 120, 150, 180, 210, and 240 min, 1 mL of the dispersion was withdrawn, centrifuged at 18,000 rpm for 2 min (Mikro 220R, Hettich, Germany), and the supernatant was UV-Vis spectroscopic analyzed at 278 nm (Jasco V730, Jasco, Japan). The percentage of CIP adsorption, as a function of time, was calculated by Eq (2). The particles after the adsorption process were then cold centrifuged at 18,000 rpm for 30 min, lyophilized (FreeZone, Labconco, USA), and kept at 4°C until uses.


$$\%\;CIP\;adsorption=\frac{Initial\;CIP\;concentration-CIP\;concentration\;at\;time\;t}{Initial\;CIP\;concentration}\times 100$$ (2)

### Particles characterizations

#### Size and zeta potential

Utilizing the zetasizer machine (SZ-100, Horiba, Japan), the particles sizes and polydispersity indexes, and zeta potentials were determined by the dynamic light scattering (DLS) and the phase analysis light scattering (PALS), respectively. For the DLS, the lyophilized particles were re-dispersed in water until a count rate of ~500 kcps, filled in a 10-mm-cuvette, and subjected to the machine measurements at 25°C using the standard settings and calibration guided by the manufacturer protocols with 10 measurement cycles/time. For the PALS, the samples were prepared similarly to that of the DLS method, and the measurement was conducted at 14.8° to the incident light. The zeta potentials were determined from the electrophoresis mobility utilizing the Smoluchowski equation.

#### Shape and morphology

The particles morphology was determined using the scanning electron microscope (SEM, JCM 7000, JOEL, Japan). The lyophilized particles were re-dispersed in water and dropped onto the SEM stub, followed by air-drying. The stub was then gold-coated and subjected to SEM observation under the nitrogen atmosphere. The images were analyzed using the ImageJ software (National Institutes of Health and the Laboratory for Optical and Computational Instrumentation (LOCI), University of Wisconsin, USA).

#### Chemical interactions and thermo-analysis

The interactions between fibroin, PVA, and CIP in the particles, as well as the changes in thermo-behaviors, were determined by the Fourier-transform infrared spectroscopy (FT-IR), X-ray diffraction (XRD), and differential scanning calorimetry (DSC) techniques.

For the FT-IR, the lyophilized particles were mixed with KBr and being pressed to form the thin pellets. The pellets were then subjected to FT-IR analysis using a spectrophotometer (Nicolet 6700, Thermo Fisher Scientific, USA), with a wavenumber range from 4,000 to 500 cm^-1^ and a resolution of 4.0 cm^-1^ in a desiccated air purge. The background signals were determined using the empty KBr pellet and removed from the sample measurement.

The XRD was performed on a Bruker D8 Advance XRD equipment (Bruker, USA), using copper K-α radiation produced at 45 kV and 36 mA. The lyophilized FNP and FNP/PVA were spread out onto quartz surfaces and examined at a scan speed of 2°/min with 2θ angles from 10 to 60°.

The DSC study was carried out using a DSC 200 F3 (Netzsch, Germany) equipped with a ceramic:FRS6 sensor. The lyophilized FNP and FNP/PVA were weighted (5–8 mg) and placed in an aluminum pan with a blank pan as a reference, and subjected to measurements with a temperature range of 40–225°C and a scanning rate of 3°C/min in a nitrogen gas flow of 20 mL/min.

#### Crystallinity

The crystallinity of the particles was determined based on the crystallinity index (i.e., the fractions of crystalline portions in a material), which could be calculated based on the correspond FT-IR spectra (Nicolet 6700, Thermo Fisher Scientific, USA) [3]. The FT-IR signal intensities of the fibroin amide-I and amide-II peaks were used in this investigation to compute the crystallinity index, following Eqs (3) and (4). The peaks of 1622 cm^-1^ (amide I) and at 1517 cm^-1^ (amide II) correspond to the crystalline portions of fibroin, and the peaks of 1646 cm^-1^ (amide I) and at 1560 cm^-1^ (amide II) correspond to the amorphous portions of fibroin.


$$Crystallinity\;index\;(amide\;I)=\frac{Intensity\;of\;the\;peak\;1622\;{cm}^{-1}}{Intensity\;of\;the\;peak\;1622\;{cm}^{-1}+Intensity\;of\;the\;peak\;1646\;{cm}^{-1}}$$ (3)

$$Crystallinity\;index\;(amide\;II)=\frac{Intensity\;of\;the\;peak\;1517\;{cm}^{-1}}{Intensity\;of\;the\;peak\;1517\;{cm}^{-1}+Intensity\;of\;the\;peak\;1560\;{cm}^{-1}}$$ (4)

### Isotherms and kinetics of CIP adsorption

To elucidate the process of CIP adsorption and its underlying mechanisms, two standard isotherm models were utilized, including the Langmuir model (Eq (5)) and the Dubinin-Radushkevich (D-R) model (Eq (6)). The Langmuir adsorption isotherm describes the equilibrium in an adsorbate-adsorbent system, where the adsorption of the adsorbate is confined to a single molecular layer, occurring at or before the relative pressure reaches unity. The D-R model characterizes the adsorption process at the solid/liquid interface, particularly for the adsorption on microporous and non-porous materials. In terms of pharmaceutical adsorption, these two models are frequently used to evaluate a drug isothermal properties [12]. The Langmuir model commonly denotes the single or multi-layer(s) drug adsorption, whereas the D-R model examines the nature of the adsorption process (physical or chemical).

$$\frac{C_{e}}{q_{e}}=\frac{C_{e}}{q_{m}}+\frac{1}{q_{m}.K_{L}}$$ (5)

$$lnq_{e}=lnq_{m}-K_{D-R}\varepsilon^{2}$$ (6)

where Ce is the equilibrium concentration of the drug CIP (mg/L), qe is the adsorption capacity of the particles (mg/g dry particles), qm is the maximum/saturated adsorption capacity (mg/g), K_L_ and K_D-R_ is the Langmuir constant (L/mg) and Dubinin-Radushkevich constant (mol^2^/kJ^2^), respectively, and $\varepsilon =RTln\;(1+\frac{1}{Ce})$is the Polanyi potential energy.

The average adsorption energy (E, kJ/mol) could be calculated from the D-R model (Eq (7)). An E value of < 8 kJ/mol indicates a physical adsorption process, whereas the chemical adsorption happens when the E values range from 8 to 16 kJ/mol.


$$E=\frac{1}{\sqrt{2\beta }}$$ (7)

Additionally, to evaluate the CIP adsorption speed and behaviors, two kinetic models were fitted, including the pseudo first-order model (Eq (8)) and the pseudo second-order model (Eq (9)).

$$ln\;(q_{e}–q_{t})=ln\;(q_{e})–k_{1}t$$ (8)

$$\frac{t}{q_{t}}=\frac{1}{k_{2}q_{e}^{2}}+\frac{t}{q_{e}}$$ (9)

where qe, qt is the CIP adsorption capacity at equilibrium time and at time t, respectively (mg/g), and k_1_, k_2_ is the first- and second-order adsorption rate constants.

### In-vitro CIP release profile

The in-vitro release profiles of CIP from FNP-CIP and FNP/PVA-CIP, for both formulation methods of desolvation and adsorption, were investigated in the simulated oral conditions. To this end, the lyophilized particles were firstly re-dispersed in 10 mL of HCl pH 1.2, simulating the gastric fluid, for 2 h. After that, the particles were continued dispersing in 30 mL of phosphate buffer pH 6.8, simulating the intestinal fluid, for another 4 h. In both media, at each time interval of 30 min, 1 mL of the mixture was withdrawn and buffer replaced. The aspirates then were centrifuged (12,000 rpm, 5 min) and the released CIP amount in the supernatant was UV-Vis spectroscopic measured (Jasco V730, Jasco, Japan). For the HCl pH 1.2, the wavelength was 277 nm, with a standard curve equation of y = 0.1065x + 0.0443, R^2^ = 0.9943. For the phosphate buffer pH 6.8, the wavelength was 271.5 nm, with a standard curve equation of y = 0.1014x + 0.0084, R^2^ = 0.9993). Finally, the percentages of cumulative CIP release were calculated using Eq (10).

$$\%\;Cumulative\;release=\frac{C_{t}V_{0}+V\sum_{1}^{t-1}C_{i}}{M_{0}-\sum_{1}^{t-1}M_{i}}\;x\;100$$ (10)

where C_t_ and C_i_ are the CIP concentrations at the time point t and i, V_0_ and V are the total volume and withdrawal volume at each time point, M_0_ and M_i_ are the initial CIP amount and the withdrawal CIP amount at the time point i.

### Antibacterial test

To evaluate the particles antibacterial ability, the in-vitro broth dilution method was conducted on 03 bacteria species of *Bacillus subtilis* (ATCC 6633, Gram (+)), *Escherichia coli* (ATCC 25922, Gram (-)), and *Salmonella enterica* (ATCC 35664, Gram (-)), following the guidelines of the European Committee on Antimicrobial Susceptibility Testing (EUCAST) [31]. The samples (the pure CIP, the blank particles, the CIP loaded particles, and the references ampicillin and cefotaxime) were serial-twofold diluted with the culture medium in a 96-well plate to get the concentration ranges from 0.25 to 1,024 μg/mL. Then, 100 μL of the bacteria suspensions (0.5 McFarland standard) in Mueller-Hinton broth (MHB) were added to each well to get a final inoculum size of 5 x 10^5^ CFU/mL, and the plates were incubated at 37°C for 24 h. The bacterial growth was determined by measuring the dispersion turbidity at 600 nm using a microplate reader (ELx808IU, Biotek, USA). The bacteria suspensions without samples served as a positive control, and the mixture of medium and the samples without bacteria was the negative control. The lowest concentrations in which the bacteria could not grow were considered as the minimum inhibitory concentrations (MIC).

### Statistical analysis

The experiments were repeated three times for each quantitative analysis, and the data was expressed as mean ± standard deviation (SD). To assess statistical significance, both the Student’s t-test and one-way analysis of variance (ANOVA) were employed, with a significance level set at 0.05 for meaningful comparisons.

## Results and discussions

Generally, the drug release rate from a carrier/material is based mostly on its interactions with the material, as well as its location on the carrier (i.e., at the carrier surface and/or in the carrier core) [32]. As such, the drug release rate from the nanoparticles’ surfaces is significantly different than that from the nanoparticles’ cores. Therefore, in this present study, we formulated the CIP loaded FNP, with or without PVA, using both the adsorption process (i.e., the drug was mainly located on the particles’ surfaces) and the desolvation process (i.e., the drug both stayed on the particles’ surfaces and in the particles’ cores). The main purpose was to determine the portions of CIP located on the FNP surfaces and in the FNP cores, linking these differences to the particle characterizations and the drug behaviors. Understanding these phenomena could be beneficial for future research on the controlled release of drugs by utilizing the drug-materials interactions.

For that reason, the blank FNP and FNP/PVA were firstly prepared with varied parameters to find the optimal formula. Then, CIP was loaded into the FNP and FNP/PVA by two different methods of desolvation and adsorption. The particles were physicochemically characterized in terms of sizes, polydispersity indexes, zeta potentials, morphologies, chemical interactions, and crystallinities. More importantly, the kinetics and mechanisms underlying the CIP adsorption/release process onto/from the particles were evaluated. Finally, the antibacterial activities of the particles were determined.

### Formulations of the blank FNP and FNP/PVA

Initially, the effects of ethanol on the FNP sizes were evaluated by varying the fibroin:ethanol ratios of 1:1, 1:2, 1:3, 1:5, and 1:7 (v/v). The results showed that the FNP sizes significantly decreased from 2,213 ± 102 nm at 1:1 ratio, to 818 ± 56 nm at 1:2 ratio, 688 ± 47 nm at 1:3 ratio, 633 ± 61 nm at 1:5 ratio, and 630 ± 44 nm at 1:7 ratio. This is explained by the self-assembly process of fibroin, which creates particles when the solvent changes from water to ethanol [3]. The fibroin molecules in the aqueous solution mainly stay in the amorphous silk-I structure with α-helix arrangements. The addition of ethanol initiates the formations of intra-/intermolecular hydrogen bonds in/amongst the fibroin molecules, thus transforming its structure to silk-II with anti-parallel β-sheet arrangements, resulting in the formation of FNP [1, 3]. Therefore, the higher the ethanol amount, the quicker the granulation process, consequently yielded smaller particles. Since the ratio of 1:5 and 1:7 formed FNP with similar sizes, the 1:5 ratio was selected for further investigations.

**Fig 1:**
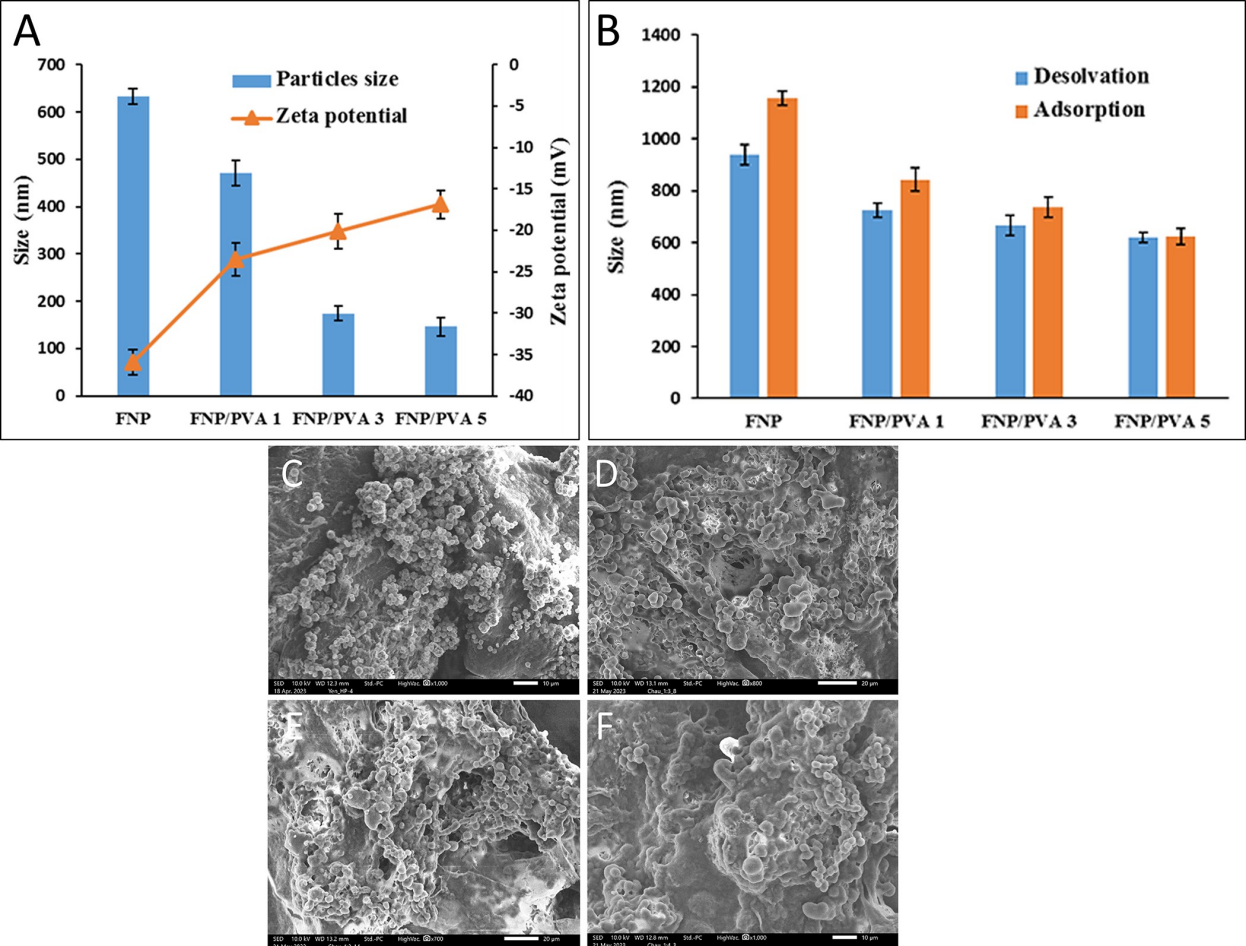
(A) Particle sizes and zeta potentials of FNP and PVA functionalized FNP (FNP/PVA) at different PVA concentrations of 1% (FNP/PVA 1), 3% (FNP/PVA 3), and 5% (FNP/PVA 5); (B) Particle sizes of CIP loaded FNP and FNP/PVA (n = 3); (C-F) SEM images of (C) FNP, (D) FNP/PVA, (E) CIP loaded FNP, and (F) CIP loaded FNP/PVA.

Next, the PVA was incorporated in the FNP matrix. PVA is a biodegradable and biocompatible semi-crystalline synthetic polymer [33] that has been utilized in the pharmaceutical industry as a solubility enhancer, coating materials, and delivery systems for hydrophilic/hydrophobic drugs [34, 35]. Theoretically, PVA steric hindrance effect could reduce the particles aggregation during the formulation process, thus making the particles smaller [36]. Moreover, PVA could form additional hydrogel bonding with the fibroin molecules, consequently covering the nanoparticles surfaces and preventing particles-particles contacts, ultimately reducing the particle sizes [37]. As expected, when the PVA concentrations increased from 0% (in the FNP) to 5% (FNP/PVA 5), the particles sizes decreased from ~630 nm to ~140 nm (**Fig 1A**). Additionally, although PVA has no charge at pH 7.0, the zeta potential absolute values of FNP/PVA decreased from ~ -35 mV in FNP to ~ -17 mV in FNP/PVA 5 (**Fig 1A**), suggesting that the PVA molecules were coated on the particles surfaces and covered the surface expressions of the negatively charged fibroin, thereby lowering the particle charges.

### Formulations of the FNP-CIP and FNP/PVA-CIP

#### Size, zeta potential, and morphology

CIP was loaded into the FNP and FNP/PVA by two different methods of desolvation and adsorption. Both techniques significantly increased the particle sizes from ~630 nm to > 1000 nm in FNP, and from ~140 nm to ~600 nm in FNP/PVA 5 (**Fig 1B**), suggesting the drug incorporations in the particle structures [8, 9]. The particle sizes of the adsorption approach were bigger than those of the desolvation method because most of the drug molecules attached to the surface of the particles, thus increasing the particle diameters. Moreover, all formulas possessed a polydispersity index of < 0.3, indicating a narrow size distribution. Additionally, the SEM micrographs (**Fig 1C–1F**) demonstrate that the blank and CIP loaded FNP and FNP/PVA were spherical with smooth surfaces, with no observable differences between the FNP and FNP/PVA, and between the blank particles and the CIP loaded ones, signifying the polymer/drug incorporating process did not alter the particle morphology.

#### Entrapment efficiency

Next, the particles were determined the EE% for the desolvation method (**Table 1**) and adsorption percentages for the adsorption method. For this, the EE% of all particles (FNP-CIP and FNP/PVA-CIP), at the same initial CIP concentration, were indifferent. On the other hand, the particles loading an initial CIP amount of 1,000 μg possessed two-fold lower the EE% than those with an initial CIP amount of 500 μg. This was due to the saturation effect. The FNP and PVA/FNP, with a total amount of 10 mg fibroin, could only produce limited interactive spaces to load a maximum amount of ~250 μg of CIP in their interior and on their surfaces, possibly via weak interactions (i.e., van der Waals forces, hydrogen bonding, and hydrophobic interactions) [8, 9]. The CIP and fibroin interactions were further discussed in the next section. As a result, a higher initial CIP amount generated a higher unloaded CIP amount (i.e., free CIP molecules that could not be bound with the FNP and FNP/PVA), consequently causing lower CIP EE%. It is worth to notice that the PVA did not help enhancing the drug EE%, although the polymer theoretically produces more interactions with CIP [38]. This phenomenon might be because PVA is also a solubility enhancer [39] that increases the CIP aqueous solubility, consequently making more CIP molecules stayed in the solution and less CIP precipitated in the particles. Conclusively, the CIP initial amount of 500 μg was deemed appropriate and cost-effective for the formulation process.

**Table 1:** Entrapment efficiency (EE%) of the CIP loaded FNP (FNP-CIP) and CIP loaded PVA functionalized FNP (FNP/PVA-CIP) (n = 3).

Sample	The initial amount of CIP (μg)	The amount of entrapped CIP (μg)	EE%
FNP-CIP	500	233.55 ± 5.40	46.71 ± 1.08
FNP/PVA 1-CIP		244.10 ± 3.45	48.82 ± 0.69
FNP/PVA 3-CIP		248.65 ± 5.35	49.73 ± 1.07
FNP/PVA 5-CIP		246.35 ± 4.95	49.27 ± 0.99
FNP-CIP	1000	290.10 ± 7.50	29.01 ± 0.75
FNP/PVA 1-CIP		208.80 ± 6.10	20.88 ± 0.61
FNP/PVA 3-CIP		265.60 ± 12.60	26.56 ± 1.26
FNP/PVA 5-CIP		205.30 ± 7.90	20.53 ± 0.79

#### Chemical interactions and thermo-analysis

**Fig 2:**
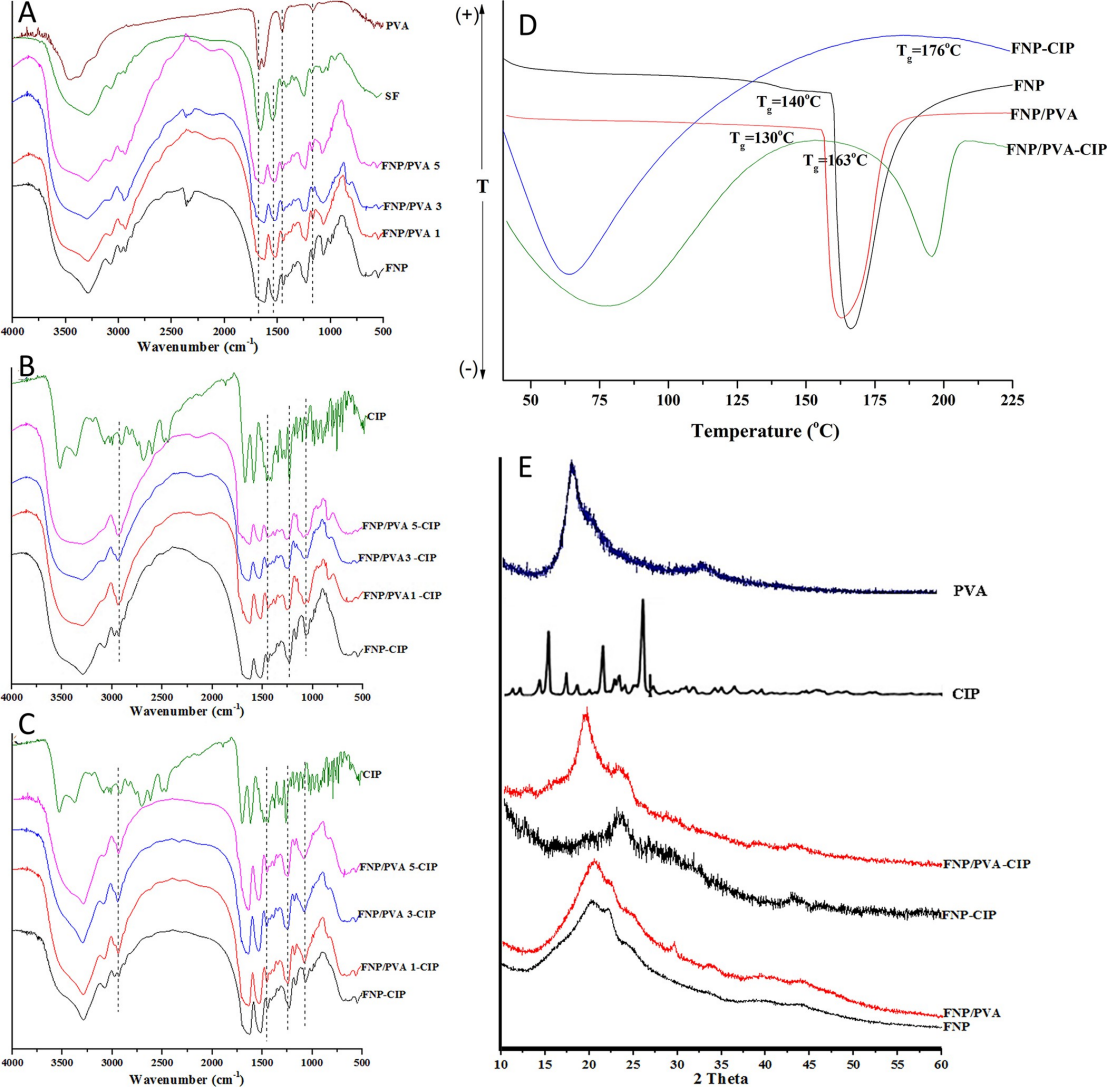
(A-C) FT-IR spectra of silk fibroin (SF), FNP, and PVA functionalized FNP (FNP/PVA) at different PVA concentrations of 1% (FNP/PVA 1), 3% (FNP/PVA 3), and 5% (FNP/PVA 5); (A) the blank particles, (B) the CIP loaded particles formulated by the desolvation method, and (C) the CIP loaded particles formulated by the adsorption method; (D) DSC graphs and (E) XRD spectra of FNP, FNP/PVA, FNP-CIP, and FNP/PVA-CIP.

In terms of the chemical interactions of the particles, FT-IR, DSC, and XRD techniques were employed (**Fig 2**). Firstly, the FT-IR spectra of the blank FNP and FNP/PVA showed the characterized fibroin signals at 1626 cm^-1^ (amide I, C = O bond), 1515 cm^-1^ (amide II, N-H bond), and 1231 cm^-1^ (amide III, C-N bond) [3, 30]. Additionally, the FNP/PVA possessed overlapped PVA signals at 3452 cm^-1^ (O-H stretching vibration), 3353 cm^-1^ (C-H alkyl bond) and an intensity increase at the 1164 cm^-1^ peak, corresponding to the PVA intra-/intermolecular hydrogen bonding (**Fig 2A**) [40, 41]. In summary, PVA was successfully incorporated in the FNP. Secondly, for the drug loaded particles, FNP-CIP and FNP/PVA-CIP, formulated by both the desolvation and adsorption methods, additional signals of CIP were observed (**Fig 2B and 2C**), including the N-H stretching vibration at 3525 cm^-1^, O-H vibration at 3373 cm^-1^, 4-quinolone ring at 1624 cm^-1^, aromatic C = C at 1495 cm^-1^, and C-F stretching vibration at 1045–1000 cm^-1^ [42]. These results suggested that CIP was successfully loaded in the particles.

Regarding the DSC thermos-analysis, the particles possessed different glass transition temperature (T_g_), followed the order of blank FNP/PVA (130°C) < blank FNP (140°C) < FNP/PVA-CIP (163°C) < FNP-CIP (176°C) (**Fig 2D**). An increase in material T_g_ is correlated with a more crystalline structure and a decrease in molecular mobility, and vice versa [43]. Therefore, the particle crystallinity could follow the same order (FNP/PVA < FNP < FNP/PVA-CIP < FNP-CIP), which was further confirmed in the **Crystallinity section**. To explain this, the PVA is an amorphous polymer with a relatively low T_g_ of 75–85°C [44], thus, when being combined with FNP, the T_g_ of the total system was reduced. On the other hand, when CIP was loaded, the particles exhibited a significantly higher T_g_, which was connected to the simultaneous crystallization of CIP (T_g_ of 168.88°C [45]) into the particle structure.

In terms of the XRD (**Fig 2E**), the FNP possessed broad amorphous peaks at the 2θ of 20° and 24°, representing the β-sheet long-range crystalline spacing of 4.5 and 3.8 Å, respectively [3]. The FNP/PVA had a small peak at 36°, indicating the characterized peak of amorphous PVA [40]. Moreover, in the CIP loaded particles, the CIP sharp signal at 25° [46] appeared in the FNP-CIP and FNP/PVA-CIP, with a small shift to around 24° due to the effects of the nearby fibroin broad peak. Additionally, the overall signal intensities in the CIP loaded particles were higher than those in the blank, which was due to the crystalline nature of CIP (confirmed in the **Crystallinity section**).

In summary, the FT-IR, XRD, and DSC both confirmed that PVA and CIP was successfully incorporated in the particles systems.

#### Crystallinity

To confirm the particle crystallinity, the FT-IR peak intensity was used for the calculation [3] (**S1 Table**). Expectedly, the crystallinity rises in the sequence of pure fibroin (0.452–0.461) < blank FNP/PVA (0.474–0.483) < blank FNP (0.503–0.517) < FNP/PVA-CIP (0.510–0.524) < FNP-CIP (0.523–0.539). This order was in total agreement with the DSC and XRD results. In summary, the pure regenerated fibroin, after the extraction process, is mostly in amorphous silk-I alpha helix structure [8]. By utilizing ethanol, a desolvation agent, this structure changes to crystalline silk-II β-sheet in FNP. The addition of PVA in the FNP reduced its crystallinity due to the inherent amorphous nature of PVA. Finally, the incorporation of the crystalline CIP enhanced the overall system crystallinity.

### Isotherms and kinetics of CIP adsorption

**Fig 3:**
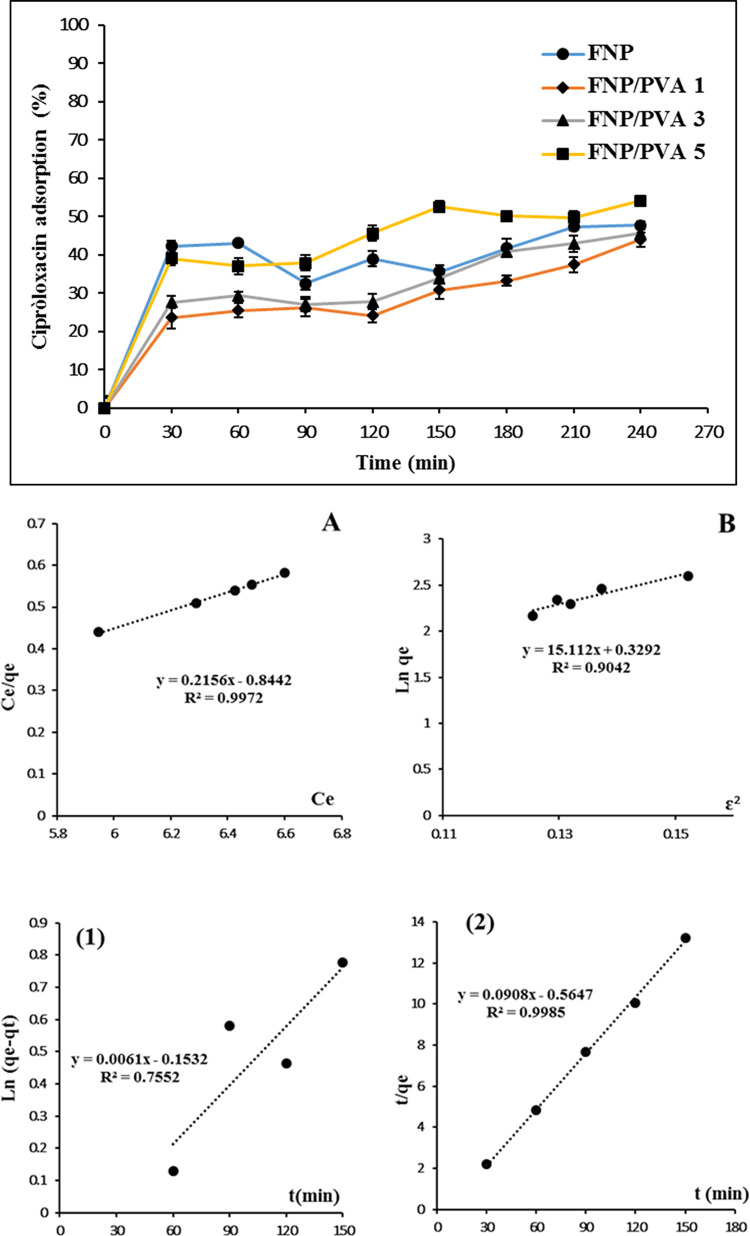
Adsorption process of CIP onto the FNP and PVA functionalized FNP (FNP/PVA) at different PVA concentrations; the particle isotherm models of (A) Langmuir and (B) Dubinin-Radushkevich; the kinetics models of (1) pseudo first-order and (2) pseudo second-order.

To deeply understand the interactions between CIP and FNP, the adsorption study was conducted. Firstly, regarding the adsorption rate, all systems could rapidly adsorb CIP within 30 min and sustain the process until 240 min, with the highest adsorption percentages of ~43% (**Fig 3**). Calculating back to the CIP amount, a maximum of ~215 μg of CIP was adsorbed on the particle surfaces. This, together with the EE% result, suggests that CIP molecules mostly interacted with the FNP surfaces (~86%), with limited molecules stayed in the particle interior (~35 μg, ~14%).

Secondly, since all particles possessed similar adsorption process, a representative formula of FNP/PVA 5 was then used for further mathematical analyses. To this end, the CIP adsorption isotherm followed the Langmuir model, with a maximum adsorption capacity of 4.64 mg/g (R^2^ = 0.9972) (**Fig 3A**), suggesting that the adsorption process was monolayer [47]. Moreover, this theoretical adsorption capacity value was less than that of the actual capacity (~21.5 mg/g), which could be because CIP was also adsorbed on the PVA molecular chains via hydrogen bonding, thus increasing the overall capacity. Additionally, the adsorption energy (E) was calculated to be 0.18 kJ/mol (< 8 kJ/mol), indicating that the adsorption process was a physical one, with weak interactions between the adsorbate (CIP) and the adsorbent (particles), including van der Waals forces, hydrogen bonding, hydrophobic interactions, and electrostatic forces. Finally, the adsorption kinetics followed pseudo second-order with regression equation of y = 0.0908x - 0.5647 (R^2^ = 0.9985) and an adsorption rate of 1.77 mg/(g.min).

Thirdly, since CIP is a zwitterionic molecule, with its charges varied dependent on the solution pH, consideration should be taken on the CIP ionization states and the correspond adsorption process. Generally, CIP has two pKa of 5.90 (carboxyl group) and 8.89 (basic N-moiety) [48]. Thus, in the adsorption environment (water) at pH 6.2, an initial CIP amount of 500 μg contained 9.39952 x 10^17^ CIP molecules, of which 39% were CIP cations and 61% were zwitterions [49, 50] (**Table 2**). On the other hand, the particles possessed a negative charge. Thus, the positively charged CIP cations were preferably adsorbed via strong electrostatic forces [51, 52], resulting in a rapid rise in adsorption efficiency in the first 30 min (**Fig 3**). After all the CIP cations were adsorbed, the particles slowly adsorbed the zwitterions via other interactions, contributing to the sustained phase until 240 min.

**Table 2:** Total number of CIP molecules and CIP charge moieties during the adsorption process.

Adsorption time	Sample	Total CIP molecules	Cation CIP molecules	Zwitterion CIP molecules
0	CIP solution	9.39952 x 10^17^	3.66581 x 10^17^	5.73371 x 10^17^
**Adsorbed CIP molecules**				
30 min	FNP	3.96660 x 10^17^	3.66581 x 10^17^	3.00785 x 10^16^
	FNP/PVA 1	2.21829 x 10^17^	2.21829 x 10^17^	0
	FNP/PVA 3	2.59427 x 10^17^	2.59427 x 10^17^	0
	FNP/PVA 5	3.66581 x 10^17^	3.66581 x 10^17^	0
240 min	FNP	3.81140 x 10^17^	3.66581 x 10^17^	2.25588 x 10^16^
	FNP/PVA 1	3.58122 x 10^17^	3.58122 x 10^17^	0
	FNP/PVA 3	3.71281 x 10^17^	3.66581 x 10^17^	4.69976 x 10^16^
	FNP/PVA 5	4.14777 x 10^17^	3.66581 x 10^17^	7.51961 x 10^16^

**Fig 4:**
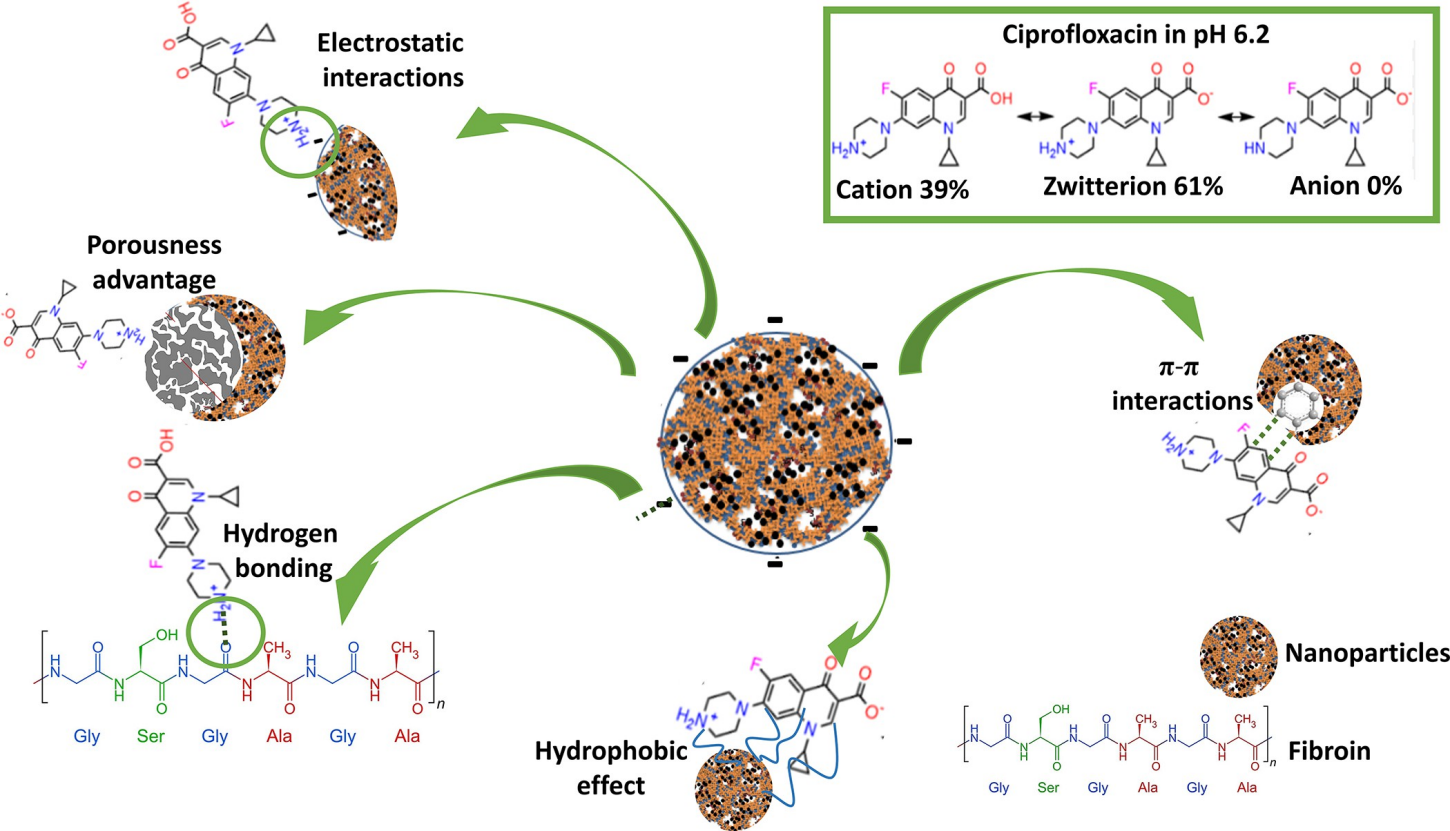
Summary of interactions between CIP and FNP during the adsorption process in water at pH 6.2.

In summary, **Fig 4** illustrates the possible molecular interactions between CIP and FNP, FNP/PVA. For this, CIP interacts with fibroin via four main forces, the electrostatic interactions, the hydrogen bonding, the hydrophobic effect, and the π-π interactions. Firstly, as previously discussed, CIP has two pKa of 5.90 (carboxyl group) and 8.89 (basic N-moiety). Thus, in water at pH 6–7, CIP mainly stays in the cationic and zwitterionic forms, making the electrostatic interactions between CIP NH_2_^+^ groups and fibroin aspartic acid/glutamic acid (COO^-^) groups. Secondly, CIP contains several functional groups capable of hydrogen bonding, including one carboxyl group, one carbonyl group, and three amino groups, which could form hydrogen bonds with fibroin hydroxyl groups (from serine or tyrosine residues), amide groups in the protein backbone, and side chain groups (i.e., carboxyl groups in aspartic acid and glutamic acid). Thirdly, hydrophobic interactions could occur between the hydrophobic fluorinated quinolone ring of CIP and the hydrophobic domains of fibroin (i.e., a fibroin molecule contains 12 hydrophobic regions with repetitive sequences of glycine, serine, and alanine, interspersed with 11 amorphous hydrophilic regions composed of random sequences [3]). Lastly, the CIP quinolone ring could form π-π interactions with benzene rings of the tyrosine residues in the fibroin molecules. These interactions strongly affect the CIP behaviors in the nanoparticles, especially its release rate, as discussed in the next section.

### In-vitro CIP release profile

**Fig 5:**
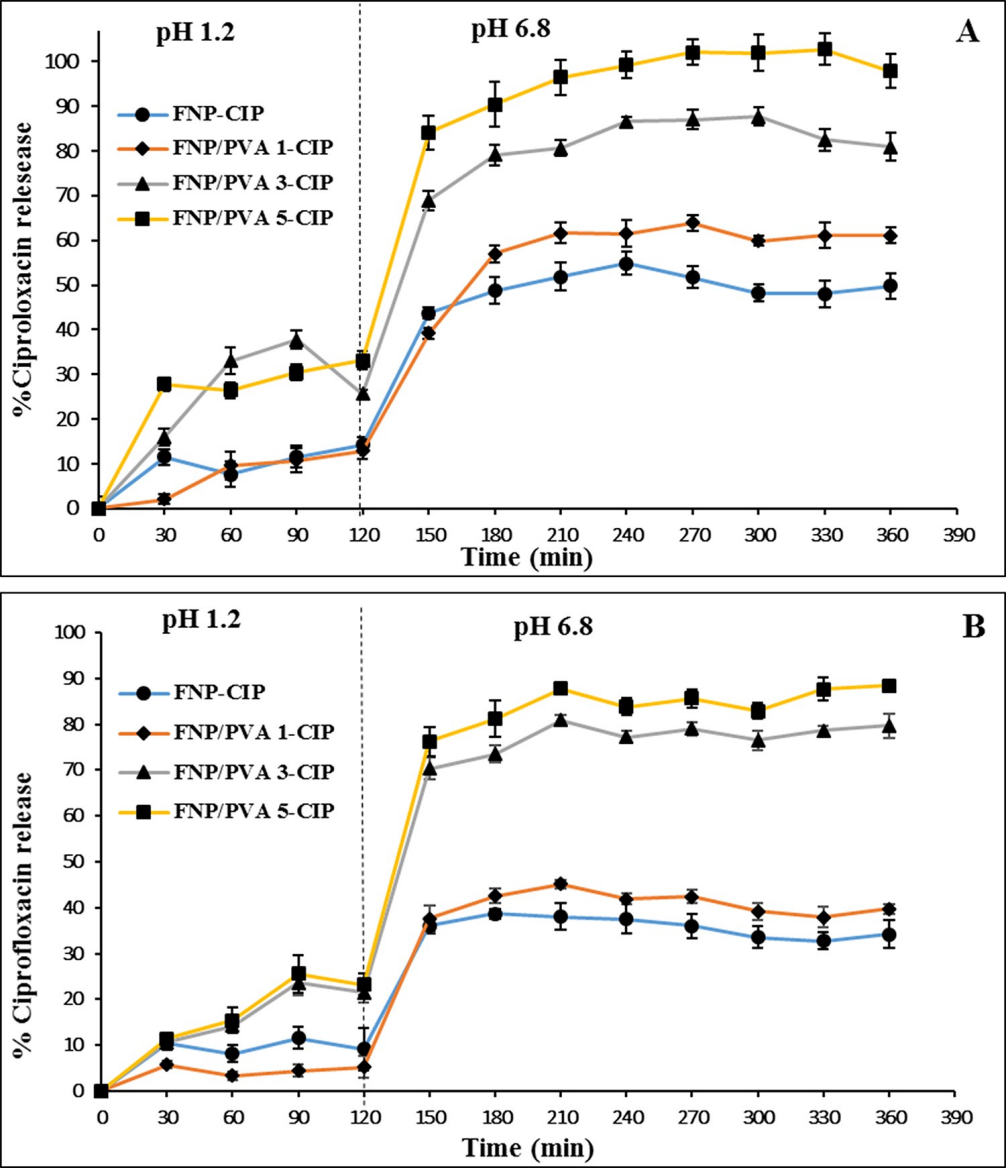
Drug release patterns, in simulated gastrointestinal condition, of CIP from the FNP-CIP and PVA functionalized FNP (FNP/PVA-CIP), formulated using (A) the desolvation method and (B) the adsorption method (n = 3).

The in-vitro release profiles of FNP-CIP and FNP/PVA-CIP were determined in the simulated gastrointestinal conditions (**Fig 5**). Firstly, particles formulated using the desolvation (**Fig 5A**) and adsorption methods (**Fig 5B**) were indifferent in terms of the release patterns, which was due to the similar drug EE% between the two methods (for desolvation, ~25 mg/g, ~86% on the particles surfaces, ~14% in the particle interior; for adsorption, ~21.5 mg/g, 100% on the particles surfaces).

Secondly, the release rates followed the order of FNP-CIP < FNP/PVA 1-CIP < FNP/PVA 3-CIP < FNP/PVA 5-CIP, possibly due to two reasons. The first reason was that the particles sizes decreased proportionally to the PVA amounts (**Fig 1B**), thus increasing the nanoparticles-medium contact areas, making more drug molecules diffused out of the particles [8, 9, 11]. The second reason was that the PVA acts as a solubility enhancer that allows more drug molecules to be released and stayed in the medium.

Thirdly and most importantly, the particles behaved differently in the simulated gastric condition (pH 1.2) and in the simulated intestinal condition (pH 6.8). Specifically, the quantity of drug released in the gastric condition within 120 min was < 40%, yet the release rates significantly increased in the intestinal condition for the remaining duration, up to ~100%, dependent on the formulations. This release rate characteristic could be beneficial for sustain release of the drug in chronic disease treatment [53]. The CIP and fibroin interactions could be used to explain this phenomenon. As previously discussed, nearly 100% of CIP molecules has a positive charge at pH 1.2 [49, 50], which strongly bound to the negatively charged particles via electrostatic interactions, consequently hampered the release process. On the other hand, at pH of 6.8, CIP molecules change their potential to a non-charged state (zwitterion), causing the electrostatic attraction between the drug and the particle to progressively decline to zero, increasing the likelihood of drug release.

In conclusion, the particles could protect the drug from the stomach acidic condition, while facilitate its release in the intestinal condition. Moreover, the oral release rates of FNP-CIP and FNP/PVA-CIP could be favorably controlled by varying the amount of PVA.

### Antibacterial test

Finally, the antibacterial efficacies of the drug loaded particles were evaluated based on the MIC values, determined as the average values of three independent experiments (**Table 3**). The blank FNP and FNP/PVA did not show any significant antibacterial actions. The pure CIP was strongly effective in all three tested bacteria strains, the *Bacillus subtilis*, *Salmonella enterica*, and *Escherichia coli*, with MIC < 0.25 μg/mL. On the other hand, the FNP-CIP and FNP/PVA-CIP possess moderate antibacterial properties, with MIC higher than that of the CIP. This was possibly due to the sustained release of CIP from these particles. Additionally, the systems were more effective at inhibiting Gram (-) bacteria (*Salmonella enterica* and *Escherichia coli*) than Gram (+) bacteria (*Bacillus subtilis*) because of the inherent strong CIP activity on Gram (-) bacteria [54].

**Table 3:** MIC values of CIP loaded FNP (FNP-CIP) and PVA functionalized FNP (FNP/PVA-CIP) on *Bacillus subtilis*, *Salmonella enterica*, and *Escherichia coli* (n = 3).

Sample	MIC (μg/mL)		
	*Bacillus subtilis*	*Salmonella enterica*	*Escherichia coli*
FNP	> 1024	>1024	> 1024
FNP/PVA	> 1024	>1024	> 1024
FNP-CIP	128	32	32
FNP/PVA-CIP	128	32	8
Pure CIP	≤ 0.25	≤ 0.25	≤ 0.25
Ampicillin	32	-	-
Cefotaxime	-	32	0.5

## Conclusions

In this study, PVA functionalized FNP containing zwitterionic CIP were successfully prepared, by both desolvation and adsorption methods, for the oral delivery. The optimal formula had a size of ~630 nm, narrow size distribution, spherical shape with smooth surface, and moderate EE% of ~50% for the desolvation method and ~43% for the adsorption method. In terms of drug-fibroin interactions, most of the drug molecules located on the particle surfaces (> 80%), mainly bound to fibroin via electrostatic forces between the positively charged CIP and the negatively charged fibroin. The drug adsorption process was the physical one, followed the Langmuir model and pseudo second-order kinetics, with in-depth discussions on the drug thermo-kinetics behaviors. In the in-vitro simulated gastrointestinal condition, the particles could protect the drug in the gastric fluid pH 1.2, with < 40% drug release, and controlled release the drug in the intestinal fluid pH 6.8, with ~100% drug release. Lastly, the products possessed adequate antibacterial activities with MIC values of 128, 32, and 8 μg/mL for *Bacillus subtilis*, *Salmonella enterica*, and *Escherichia coli*, respectively. Although the study shows potential, its limitation is that it was conducted in in-vitro settings, thus lacking data from in-vivo experiments. Additionally, this work employed the fibroin extracted from M45 silkworm species, which might be different with other species. Future research on animal models and other silkworm species could address these challenges. Conclusively, the FNP could be a potential oral delivery system for zwitterionic drugs that favorably controls the drug properties via drug-carrier interactions.

## Supporting information

S1 Table Crystallinity index of the silk fibroin (SF), blank FNP, PVA functionalized FNP (FNP/PVA), and CIP loaded particles. (DOCX)

S1 Graphical abstract (TIF)
