# Correction: Formulations of poly(vinyl alcohol) functionalized silk fibroin nanoparticles for the oral delivery of zwitterionic ciprofloxacin

**DOI:** 10.1371/journal.pone.0348538

**Published:** 2026-05-07

**Authors:** Nguyen Thi Phuong Thao, Ngoc Yen Nguyen, Van Ben Co, Luong Huynh Vu Thanh, Manh Quan Nguyen, Suchiwa Pan-On, Duy Toan Pham

There are similarities between some FT-IR spectra reported in Fig 2 of this article [1] and those published in other articles by some of the same authors. Specifically:

- The PVA spectrum in Fig 2A of [1] appears similar to the PVA spectrum in Fig 5 of [2];
- The SF spectrum in Fig 2A [1] appears similar to the SF spectrum in Fig 4 of [3] and the silk fibroin spectrum in Fig 5 of [4];
- The FNP spectrum in Fig 2D of [1] appears similar to the FNP spectrum in Fig 5C of [4].

With this notice, the authors clarify that the similar FT‑IR spectra reflect the use of the same compounds, which served as controls or base materials in the experiments.

In addition, contrary to the Data Availability statement in [1], the individual-level underlying data supporting the article’s results were not provided. The quantitative data underlying Figs 1, 3, 5, and Tables 1-3 are provided with this notice as S1-S6 Files.

## Supporting information

S1 File Dataset supporting particle size and zeta potential measurements presented in Figs 1A and B. (XLSX)

S2 File Dataset for the ciprofloxacin (CIP) absorption data and fitted model shown in Fig 3. (RAR)

S3 File Dataset for CIP release profiles reported in Fig 5. (XLSX)

S4 File Data used for the analysis of CIP entrapment efficiency in Table 1. (XLSX)

S5 File Dataset underlying the analysis of CIP ionisation states in Table 2. (XLSX)

S6 File Data supporting the antimicrobial activity analysis presented in Table 3. (PDF)

## References

[1] Thi Phuong Thao N, Nguyen NY, Co VB, Thanh LHV, Nguyen MQ, Pan-On S, et al. Formulations of poly(vinyl alcohol) functionalized silk fibroin nanoparticles for the oral delivery of zwitterionic ciprofloxacin. PLoS One. 2024;19(8):e0306140. doi: 10.1371/journal.pone.0306140 39088490 PMC11293643

[2] Pham DT, Nguyễn NY, Nguyễn QCT, Quách THD, Hà TKQ. Bào chế hệ vi hạt từ fibroin tơ tằm phối trộn polymer định hướng ứng dụng dẫn truyền thuốc đường uống. *Tạp chí Khoa học Đại học Cần Thơ* CTU J Sci. 2024;60(4):48–54. doi: 10.22144/ctujos.2024.395

[3] Nguyen TTN Phuong, Nguyen NY, Cang TH Nhu, Nguyen KA, Nguyen M Thai, Nguyen HG Phat, Pham DT. Developments and characterizations of silk fibroin masks containing antibacterial allicin. TNU Journal of Science and Technology. 2024;229(2):68–75. doi: 10.34238/tnu-jst.9515

[4] Pham DT, Nguyen DXT, Nguyen NY, Nguyen TTL, Nguyen TQC, Tu AVT, et al. Development of pH-responsive Eudragit S100-functionalized silk fibroin nanoparticles as a prospective drug delivery system. PLoS One. 2024;19(5):e0303177. doi: 10.1371/journal.pone.0303177 38781182 PMC11115272

