# Supplementary material for: Formulations of poly(vinyl alcohol) functionalized silk fibroin nanoparticles for the oral delivery of zwitterionic ciprofloxacin
Source: PLoS One. 2024 Aug 1;19(8):e0306140. doi: 10.1371/journal.pone.0306140 (PMC11293643; doi:10.1371/journal.pone.0306140)
Supplement: S1 Table — (DOCX) [file pone.0306140.s001.docx]

**Table S1. Crystallinity index of the silk fibroin (SF), blank FNP, PVA functionalized FNP (FNP/PVA), and CIP loaded particles.**

| Samples | Crystallinity index | |
| --- | --- | --- |
|  | Amide I | Amide II |
| SF | 0.452 | 0.461 |
| FNP | 0.517 | 0.503 |
| FNP/PVA 1 | 0.483 | 0.478 |
| FNP/PVA 3 | 0.481 | 0.474 |
| FNP/PVA 5 | 0.480 | 0.475 |
| FNP-CIP | 0.539 | 0.523 |
| FNP/PVA-CIP | 0.524 | 0.510 |
