# Supplementary figures and images for: Formulations of poly(vinyl alcohol) functionalized silk fibroin nanoparticles for the oral delivery of zwitterionic ciprofloxacin
Source: PLoS One. 2024 Aug 1;19(8):e0306140. doi: 10.1371/journal.pone.0306140 (PMC11293643; doi:10.1371/journal.pone.0306140)

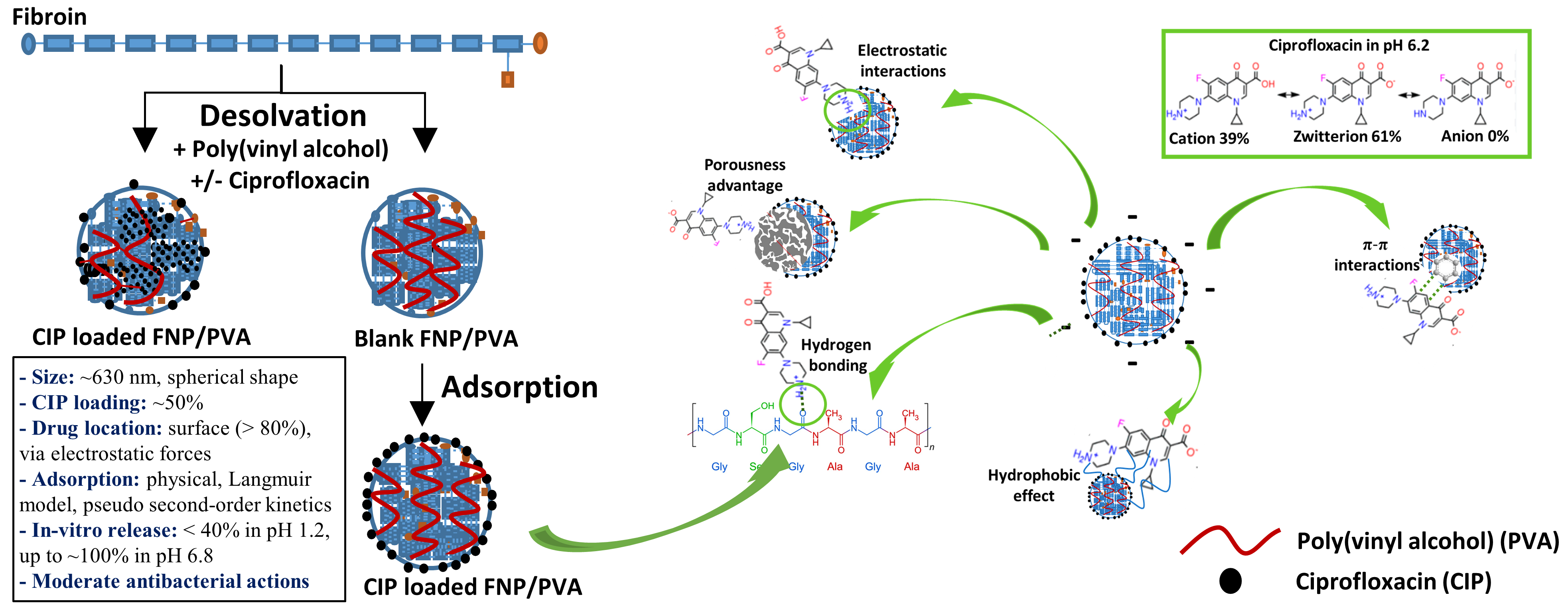

Supplement: S1 Graphical abstract — (TIF) [file pone.0306140.s002.tif]
