# Supplementary material for: Correction: Formulations of poly(vinyl alcohol) functionalized silk fibroin nanoparticles for the oral delivery of zwitterionic ciprofloxacin
Source: PLoS One. 2026 May 7;21(5):e0348538. doi: 10.1371/journal.pone.0348538 (PMC13152144; doi:10.1371/journal.pone.0348538)
Supplement: S6 File — (PDF) [file pone.0348538.s006.pdf]

VIỆN HÀN LÂM KHOA HỌC & CÔNG NGHỆ VIỆT NAM
VIỆN HOÁ HỌC
PHÒNG HÓA SINH ỨNG DỤNG
Tel: (+84) 24-37914586
Phòng 601, Nhà A18, 18 Hoàng Quốc Việt, Cầu Giấy, Hà Nội, Việt Nam

PHIẾU KẾT QUẢ THỬ HOẠT TÍNH KHÁNG SINH

Người gửi mẫu: Nguyễn Ngọc Yến, Đh Cần Thơ

Ngày gửi mẫu: 032/2023 Số lượng: 05 mẫu

**TT**	**Tên mẫu**	**MIC**						
		Gram (+)			Gram (-)			Nấm
		Staphylococcus aureus	***Bacillus subtilis***	***Lactobacillus fermentum***	***Salmonella enterica***	***Escherichia coli***	***Pseudomonas aeruginosa***	***Candida albican***
1	FNP	>1024	>1024	>1024	>1024	>1024	>1024	>1024
2	FNP/PVA	>1024	>1024	>1024	>1024	>1024	>1024	>1024
3	FNP-CIP	>1024	**128,0±0,0**	>1024	**32,0±0,0**	**32,0±0,0**	>1024	>1024
4	FNP/PVA-CIP	>1024	**128,0±0,0**	>1024	**32,0±0,0**	**8,0±0,0**	>1024	>1024
5	CIP	**≤0,25**	**≤0,25**	>128	**≤0,25**	**≤0,25**	>128	>128
Chất tham chiếu	Ampicillin	0,125±0,0	32±0,0	32±0,0				
	Cefotaxime				32±0,0	0,5±0,0	8±0,0	
	Nystatin							8±0,0

Hà Nội, ngày 17 tháng 02 năm 2023
